# Quantum dots to probe temperature and pressure in highly confined liquids

**DOI:** 10.1039/c8ra03652g

**Published:** 2018-06-21

**Authors:** Sayed M. B. Albahrani, Tarek Seoudi, David Philippon, Lionel Lafarge, Peter Reiss, Hamza Hajjaji, Gérard Guillot, Michel Querry, Jean-Marie Bluet, Philippe Vergne

**Affiliations:** Univ Lyon, INSA Lyon, CNRS, LaMCoS, UMR5259 F-69621 Villeurbanne France david.philippon@insa-lyon.fr; Univ. Grenoble Alpes, CEA, CNRS, INAC-SyMMES-STEP 38000 Grenoble France; Univ Lyon, INSA Lyon, CNRS, INL, UMR5270 F-69621 Villeurbanne France

## Abstract

A new *in situ* technique for temperature and pressure measurement within dynamic thin-film flows of liquids is presented. The technique is based on the fluorescence emission sensitivity of CdSe/CdS/ZnS quantum dots to temperature and pressure variations. In this respect, the quantum dots were dispersed in squalane, and their emission energy dependence on temperature and pressure was calibrated under static conditions. Temperature calibration was established between 295 K and 393 K showing a temperature sensitivity of 0.32 meV K^−1^. Pressure calibration was, in turn, conducted up to 1.1 GPa using a diamond anvil cell, yielding a pressure sensitivity of 33.2 meV GPa^−1^. The potential of CdSe/CdS/ZnS quantum dots as sensors to probe temperature and pressure was proven by applying the *in situ* technique to thin films of liquids undergoing dynamic conditions. Namely, temperature rises have been measured in liquid films subjected to shear heating between two parallel plates in an optical rheometer. In addition, pressure rises have been measured in a lubricated point contact under pure rolling and isothermal conditions. In both cases, the measured values have been successfully compared with theoretical or numerical predictions. These comparisons allowed the validation of the new *in situ* technique and demonstrated the potential of the quantum dots for further mapping application in more complex and/or severe conditions.

## Abstract 2

A new *in situ* technique using CdSe/CdS/ZnS quantum dots fluorescence to probe pressure and temperature within highly confined flows of liquids.

## Graphical abstract

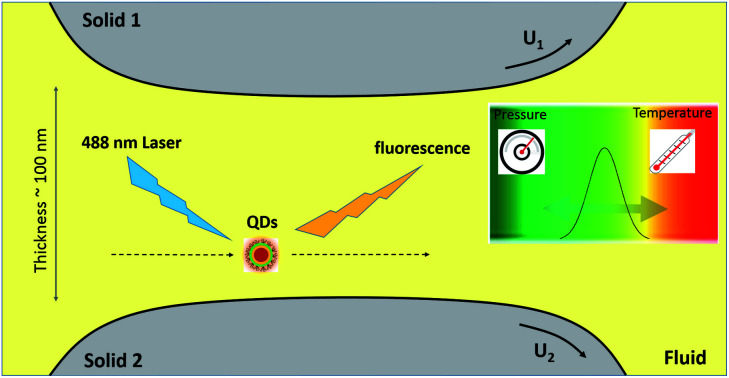

## Introduction

Very thin liquid films are formed in the so-called elastohydrodynamic lubrication (EHL) regime. This regime occurs within non-conforming contacts encountered in many machine components such as gears, bearings and cams and followers. The operating conditions (high load, high specimen velocity) as well as the geometry of such contacts lead to the concentration of extreme physical conditions within a very small area (∼400 μm in diameter). Typically, the lubricant is subjected to high pressures (1–3 GPa) and high shear rates (up to 10^6^ to 10^7^ s^−1^) resulting in large elastic deformation of the contacting surfaces, important lubricant viscosity increase and high shear stresses. Film thickness and friction are usually considered as the key parameters to assess the working conditions of elastohydrodynamic (EHD) contacts. The lubricant film thickness lies in the range of 50 to 500 nm: a thicker film would be accompanied by an increase of friction and temperature, resulting in a loss of performance. On the other hand, insufficient lubricant film thickness would not ensure a full separation between the surfaces as needed to prevent interactions between asperities and thus wear.

Film thickness and friction are determined by the rheological properties of the lubricant and the contact parameters in two distinct regions: the inlet and the high-pressure central zones, respectively. The rheological properties at each point of the contact are, in turn, inextricably related to the local temperature and pressure. The precise mapping of these latter is thus of crucial importance for improving our knowledge on the actual conditions occurring in such confined interfaces, for the validation of numerical models in the case of severe operating conditions and eventually for extending the lifetime and optimizing the performance of tribological systems.

Numerical models based on the elasticity, film thickness and Reynolds equations applied on elastic solids and piezo-viscous liquids are used to predict lubricant film thickness, and were successfully applied under many operating conditions.^1,2^ At high shear rates, however, viscous friction and shear heating can be important and the application of these models becomes more critical owing to the importance of these effects. This is because of the significant influence of temperature on film thickness generation through the dependence of fluid properties to temperature, and the lack of experimental data on temperature distribution, especially at the inlet of the contact. When it comes to predicting friction, the current knowledge on the mechanisms behind it is relatively poor because the extreme conditions at the central region involve a more complex rheology (high-pressure, high shear stress). The investigation of such phenomena and mechanisms in realistic conditions by reproducing such extreme conditions in a controlled way outside the contact is not always possible. Consequently, a reliable prediction and ultimately a good control of both film thickness and friction rely on the accurate knowledge of physical parameters, in particular temperature and pressure, throughout the contact area.

In this respect, various *in situ* techniques have been developed for the measurement of temperature and pressure in EHL. The most relevant examples are electrical resistance,^3–5^ Infrared (IR) thermography^6–8^ and Raman spectroscopy.^9–13^

The first technique takes advantage from the electrical resistance sensitivity of some materials, such as manganin®, titanium or platinum, to pressure and temperature variations. A thin transducer (0.03 to 0.15 μm thick) manufactured with such materials is deposited on one of the rubbing surfaces and is used to obtain the pressure or temperature profiles across the contact. This technique is particularly suitable for investigating real machine components since it does not require a transparent surface. Using electrical transducers has nevertheless some disadvantages. These transducers are indeed intrusive regarding the typical EHD film thickness range (50–500 nm), and they may be vulnerable with regard to the extreme conditions occurring in EHD contacts, especially in the presence of high shear stresses.^14^

In the second technique, the total IR emission emanating from the contact is collected. Conveniently, it is possible to map temperature all over the contact area by using a full-field IR camera. A drawback of this technique is the necessity of using a specimen transparent to the employed radiation, which is a common limitation in all optical *in situ* methods. The other drawback relies in the calibration process. Indeed, different sources (solid surfaces and bodies, lubricant film and the environment) contribute to the overall IR radiation, and they have to be separated by applying appropriate coatings on the transparent disc and/or using different filters.

The third technique, Raman micro-spectroscopy is a powerful tool for local measurements in EHD contacts. The Raman spectrum is representative of the vibrational state of the lubricant and yields qualitative and quantitative information about its chemical structure and bonds. Physical parameters such as film thickness, pressure and temperature can be measured by determining, respectively, the Raman peaks intensity, the peak shifts and the relative Stokes/anti-Stokes peaks. This technique presents an appropriate spatial resolution unlike IR thermometry. Jubault *et al.* published several studies in which pressure mapping was successfully achieved in EHD contacts.^11–13^ The main drawbacks of this technique are the extremely low signal-to-noise ratio, and the need to select a fluid which is a strong Raman scatterer. For more information about *in situ* techniques applied to lubrication, their advantages and limitations, interested readers can refer to the reviews by Spikes^15^ and more recently by Albahrani *et al.*^16^

The emergence of nanotechnologies in many scientific and technological fields offers an interesting alternative for the measurement of pressure and temperature in highly confined liquids. Many luminescent nanothermometers have been proposed in the literature, such as fluorescent semiconductor nanoparticles (also called quantum dots or QDs),^17–20^ fluorescent dyes,^21^ rare-earth ions^22,23^ and polymers.^24^ These nanothermometers are based on the temperature dependence of luminescence features (intensity, band shape, peak position, peaks intensity ratio or lifetime), and have been applied in many fields including micro/nano electronics, integrated photonics and biomedicine.^25^ Less studied in the literature, some luminescent nanomaterials may also be sensitive to pressure and viscosity, in particular quantum dots^26^ and molecular rotors,^27^ respectively.

In this context, the present paper aims to demonstrate the potentiality of CdSe/CdS/ZnS core/shell/shell QDs as temperature and pressure nanosensors for application in highly confined liquids, and especially in the very thin films found in EHD contacts. For this purpose, the variations in fluorescence emission with temperature and pressure of quantum dots dispersed in squalane were calibrated under static conditions. The temperature sensitivity was then used to evaluate the self-heating induced within a thin liquid film subjected to high shear rates in an optical rheometer. Finally, the pressure sensitivity was in turn used to achieve *in situ* measurements of pressure at the center of EHD point contacts.

## Experimental

### Materials

The used CdSe/CdS/ZnS QDs are constituted of a core of CdSe nanocrystal, encapsulated within two shells of semiconductor materials (CdS and ZnS), themselves covered by an organic ligand (stearic acid). The role of the intermediate CdS shell is that of a “crystal lattice adapter”, reducing the strain between the CdSe core and ZnS outer shell. More details about the synthesis, structure and optical properties of CdSe/CdS/ZnS QDs can be found elsewhere.^28,29^ The CdSe/CdS/ZnS QDs were dispersed at a concentration of 0.125 mg ml^−1^ in squalane (C_30_H_62_, supplied by Sigma-Aldrich, 99%) for subsequent calibration (temperature and pressure) and validation tests (shear heating and pressure measurements). The rheological and thermal properties of squalane are listed in Table 1.

**Table 1:** Rheological and thermal properties of squalane. The thermal-viscosity coefficient is given for two expressions of the viscosity–temperature dependence: *μ* = *μ*_0_ exp(*β*_1/*T*_(1/*T* − 1/*T*_0_)) and *μ* = *μ*_0_ exp(*β*_*T*_(*T* − *T*_0_))

Temperature	293.15	K
Low shear viscosity *η*_0_	34.34	mPa s
Pressure–viscosity coefficient α*	21.0	GPa^−1^
Thermal conductivity *k*	0.12 (ref. 30)	W m^−1^ K^−1^
Thermal-viscosity coefficient *β*_1/*T*_	4063	K
Thermal-viscosity coefficient *β*_*T*_	0.047	K^−1^

### Temperature cell

For temperature calibration, a droplet of 10 μl of the sample (squalane + QDs) was deposited on an aluminum holder under a 170 μm thick cover-glass plate, with the latter being glued on the former. The sample holder was put inside a heating ring. Teflon insulators were used to enclose the system and improve the thermal regulation. A thermocouple was used and the temperature was regulated close to the sample with a precision of +/−0.1 K between 293 K and 373 K.

### Rheometer

An optical rheometer (Physica MCR301, Anton Paar) with a parallel-plates geometry was used for fluid viscosity measurements within a max. 5% uncertainty and for nanothermometry application under dynamic conditions. For the latter, a steel plate was rotated against a fixed glass plate with a gap of 0.1 mm. The glass plate was employed for observation purpose and in order to perform spectroscopic measurements. For mechanical resistance issues, the glass plate was supported by a steel part in which an oblong groove has been machined. A shear rate in the fluid sample up to 50 000 s^−1^ was achieved during the experiments. This value was obtained at a distance of 2/3*R* from the axis of rotation, with *R* being the radius of the rotating disc, at a location that corresponds to the optical passage, *i.e.* in front of the groove made in the steel support. A Peltier device was used for heating or cooling the sample, together with a platinum sensor allowing temperature stabilization with a precision of +/−0.03 K between 293 K and 373 K.

### High pressure cell

Pressure calibrations were conducted using a high pressure diamond anvil cell (DAC). In this work, experiments were conducted up to 1.2 GPa at two different temperatures (296 and 323 K), in a DAC designed for low–medium pressure experiments (MDAC type BHP for Biology 100° symmetrical aperture, Betsa). In this cell, the sample is deposited inside a nickel gasket compressed in between a 1.4 mm culet diamond anvil and a 400 μm thick diamond window. The nickel gasket thickness was about 0.2 mm, and comprises a hole of 0.5 mm in diameter. Pressure was increased and maintained in the cell by inflating an internal membrane with helium. Pressure inside the cell was determined with a precision of ±50 MPa from the calibrated shift^31^ of the R1 fluorescence line of ruby microspheres added in the sample. In order to ensure the accuracy of the measurements and an equilibrium state of the sample, ruby luminescence shifts were checked before and after each QDs spectrum recording. A thermocouple was used to measure the temperature close to the sample with a precision of +/−0.1 K.

### EHD tribometer and operating conditions

Pressure measurements by fluorescence were carried out with a ball-on-disc tribometer. The ball and the disc were independently driven by two motors to produce pure rolling conditions. Fluorescence measurements require a transparent substrate able to transmit visible light. The contact was thus performed between a steel ball (radius of 12.7 mm) and a transparent (glass or sapphire, radius of 42.5 mm, thickness of 10 mm) disc: the material properties are given in Table 2. The roughness of the ball and the discs surfaces were smaller than 5 and 10 nm, respectively. Fully flooded contact conditions were insured by the ball that dips into the lubricant reservoir. In this test-rig, the two specimen and the lubricant are thermally isolated and maintained at constant temperature by an external thermal controlling system. A platinum probe located underneath the ball monitors the contact inlet temperature within +/−0.1 K.

**Table 2:** Mechanical and thermal properties of the contacting materials

	*E* (GPa)	*ν* (—)	*ρ* (kg m^−3^)	*κ* (W m^−1^ K^−1^)	*C*_p_ (J kg^−1^ K^−1^)
Steel ball	210	0.3	7850	46	470
Glass disc	81	0.208	2510	1.114	858
Sapphire disc	360	0.34	4000	40	750

The operating conditions are summarized in Table 3. The entrainment velocity *u*_e_ was chosen at the lowest to limit shear heating at the contact inlet, but high enough to ensure a sufficient film thickness (central film thickness *h*_c_ > 200 nm) in order to collect an adequate fluorescence signal. The central film thickness was calculated using the Chittenden formula.^32^ One temperature (293 K) and four normal loads (from 25 to 75 N) were selected leading to Hertzian pressures at the contact center from 0.5 to 1.1 GPa. Thus, a glass disc was used for medium contact pressures (0.5–0.7 GPa) and a sapphire disc for pressures around 1 GPa. The film thickness thermal reduction coefficient proposed by Cheng,^33^*ϕ*_*T*_ was calculated for each experiment. Jubault *et al.*^13^ and later Chaomleffel *et al.*^34^ showed that shear heating occurs at the contact inlet when *ϕ*_*T*_ < 0.96. This was found to be the case in our experiments. It was thus considered that the liquid flow remained isothermal.

**Table 3:** Operating conditions and predicted central film thickness and pressure at *x* = 0

Disc	Glass				Sapphire			
Temperature *T* (K)	293							
Load *w* (N)	28		75		28.5		52	
Hertzian pressure (GPa)	0.505		0.701		0.905		1.106	
Contact radius *a* (mm)	0.163		0.226		0.123		0.150	
Speed *u*_e_ (m s^−1^)	1.5	0.7	1.5	0.7	1.5	0.7	1.5	0.7
*ϕ*_*T*_ from Cheng^33^	0.97	0.99	0.97	0.98	0.97	0.99	0.97	0.99
*h*_c_ (nm) from Chittenden *et al.*^32^	362	216	337	201	327	195	313	186
Calculated *h*_c_ (nm)	370	222	346	204	333	203	319	195
Calculated pressure at *x* = 0 (GPa)	0.513	0.516	0.710	0.713	0.929	0.940	1.129	1.138

### EHD numerical solutions

The numerical model used here to predict the film thickness and the pressure profiles in the EHD contacts has been already detailed by Wheeler *et al.*^35^ This model was derived from the one developed by Doki-Thonon *et al.*^36^ who were inspired by the works of Habchi *et al.*^37,38^ It was applied here to solve the steady-state EHD problem under isothermal, Newtonian and fully flooded conditions, assuming smooth surfaces. Based on the finite element method, the numerical model solves simultaneously the generalized Reynolds, the solids deformation and the load balance equations. The model includes the modified WLF-Yasutomi correlation for the temperature–pressure–Newtonian viscosity dependence^39^ and the Tait equation^40^ for the density variations.^41^ Both equations were fitted to independent characterizations carried out with high-pressure devices.^39^ This EHL solver has been successfully validated by quantitative comparisons with experiments performed under various conditions.^36–38^ The pressure and film thickness values at the contact center calculated for the operating conditions detailed previously are reported in Table 3 and are referred to as calculated *h*_c_ and calculated pressure at *x* = 0.

### Fluorescence measurements

All the experimental setups previously described were operated together with an optical system comprising a laser source, a microscope and a spectrometer. Fluorescence measurements were carried out with an U1000 Horiba Jobin-Yvon spectrometer coupled by an optical fiber to a dedicated microscope. The light captured by the later was dispersed *via* a double monochromator with 100 gr/mm gratings, coupled with a high sensitivity 2000 × 800 CCD detector, yielding a resolution of 0.07 nm per pixel (0.20 meV per pixel). A continuous argon-ion laser with an excitation wavelength of 488 nm and a power of 1 mW on the sample (excitation density of 300 W cm^−2^ approximately) was used. Measurement in the rheometer were made with a 20× objective resulting in a measured laser spot diameter of 20 μm on the sample. Measurements during EHD tests were made with a 50× objective leading to a measured laser spot diameter of 10 μm in the contact area (excitation density of 1200 W cm^−2^ approximately). In order to perform measurements at any point within the region of interest (contact area, DAC hole, rheometer gap), the fluorescence microscope can be precisely moved and positioned above the setups by means of a motorized micro-positioning XY system. The Z displacement is used to focus the laser and the fluorescence collection from the sample. In order to remove wavelength-dependent instrumental undesirable effects, in particular slight room temperature variations, all spectra were corrected using the characteristic lines of a neon spectral calibration lamp. For each spectrum, a Gaussian distribution was used to fit the energy spectrum and determined the energy at maximum emission (*E*_emission_) of the QDs.

## Results and discussions

### Principle of temperature and pressure measurement using photoluminescence of quantum dots

Photoluminescence is the emission of light from a given substance occurring after the absorption of photons (optical radiation). Photoluminescence processes are divided in two categories: fluorescence and phosphorescence. In general, fluorescence is “fast” (1–10 ns) while phosphorescence is “slow” (longer time scale, 1 ms to 100 s). The characteristics of the emitted photons are highly affected by the interaction of the photoluminescent substances during their exited state with their local environment. Therefore, the properties of the local environment, including the polarity and the viscosity of the solvent, the local temperature and pressure can affect the emission features. Various parameters of luminescence can be analyzed including the intensity, the spectral position, the bandwidth, the lifetime, the polarization and the band-shape. For more information about fluorescence, the interested reader can refer to dedicated literature.^42^

In semiconductor materials, light absorption above band gap results in the creation of an electron–hole pair. Electron moves from the valence band into the conduction bands, leaving a hole behind. The relaxation of the exciton (electron–hole pair) is accompanied with a light emission of energy corresponding to the intrinsic semiconductor bandgap energy (*E*_g_) decreased by the coulombic interaction energy between the electron and the hole.

Quantum dots are fluorescent nanosized crystals made of semiconductor materials. QDs have been first discovered by Ekimov *et al.*,^43^ and exhibit the so-called quantum confinement effect induced by their nanometric size. This effect modifies profoundly the electronic structure of these objects when compared to a bulk semiconductor material. Indeed, QDs possess atomic-like, discrete energy levels instead of the continuous conduction and valence bands observed in their corresponding bulk materials. In addition, the band gap is strongly dependent on the size of the QDs. Thus, a wide range of emission wavelengths is available by changing their size. QDs provides several favorable spectral features: among them, the high emission efficiency, the narrow and roughly symmetrical emission spectrum (no long-wavelength tail common to all fluorophores) can be mentioned. Hence, QDs provide a variety of application in optoelectronic devices such as photovoltaic cells,^44^ light emitting diodes or LEDs^45,46^ and QD lasers.^47^ QDs are also employed for various sensing applications. For instance, they are used as biological labels for *in vivo* imaging,^48,49^ temperature probes^50–53^ and strain gauges.^26^

The Effective Mass Approximation (EMA) is used to describe the size-dependence of the bandgap energy. This approach, firstly proposed by Efros^54^ and later modified by Brus,^55^ stipulates that the bandgap of QDs is shifted from their corresponding bulk material *E*^B^_g_ with a quantity *E*^Q^_g_ arising from the (quantum) confinement:

1 *E*_g_ = *E*^B^_g_ + *E*^Q^_g_(*r*)

The dependence of the bandgap energy of semiconductor bulk materials *E*^B^_g_ to temperature is a well-documented phenomenon. These variations arise essentially from the influence of the crystalline lattice of semiconductor materials and the interaction of the exciton with the lattice. More precisely, a major contribution arises from the temperature dependence of the electron–lattice interaction, and a minor contribution arises from the shift in the relative position of the valence and conduction bands due to the lattice thermal dilatation.

The sensitivity to temperature is commonly described by the Varshni law:^56^2
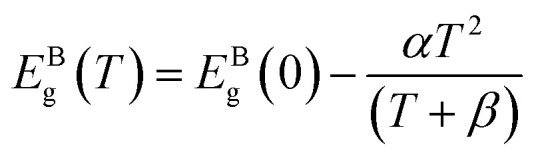
where *E*^B^_g_(0) is the bandgap energy at 0 K and 0.1 MPa, *α* is a constant and *β* is a temperature close to the Debye temperature *θ*_D_.

Theoretical treatments show linear effect with temperature at high temperature, in particular temperatures higher than the Debye temperature.

3 *E*^B^_g_(*T*) = *E*^B^_g_(0) + *α*_1_*T*

where *α*_1_ is constant.

Unlike bulk bandgap energy (*E*^B^_g_), the dependence of the quantum-confined energy (*E*^Q^_g_) to temperature is less documented in the literature. These variations arise from the size-dependence (thermal expansion) of the QDs and the temperature-induced change in the electron–phonon coupling energy.^25^ According to previous works,^57,58^ the magnitude of the quantum contribution is minor compared to the bulk contribution. Therefore, Varshni law can be used to describe the fluorescence temperature dependence of QDs.

The variation of the energy gap with pressure results from the shift in relative position of the valence and conduction bands due to the lattice compressibility and the pressure dependence of the electron lattice interaction. Linear and quadratic variations for the *E*_g_ are commonly used, for either bulk^59,60^ and nanosized semiconductors:^61^

4 *E*^B^_g_(*P*) = *E*^B^_g_(0) + *α*_2_*P*

5 *E*^B^_g_(*P*) = *E*^B^_g_(0) + *α*_2_*P + β*_2_*P*^2^

where *α*_2_ and *β*_2_ are constants.

### Appropriate selection of quantum dots and fluid

**Fig. 1:**
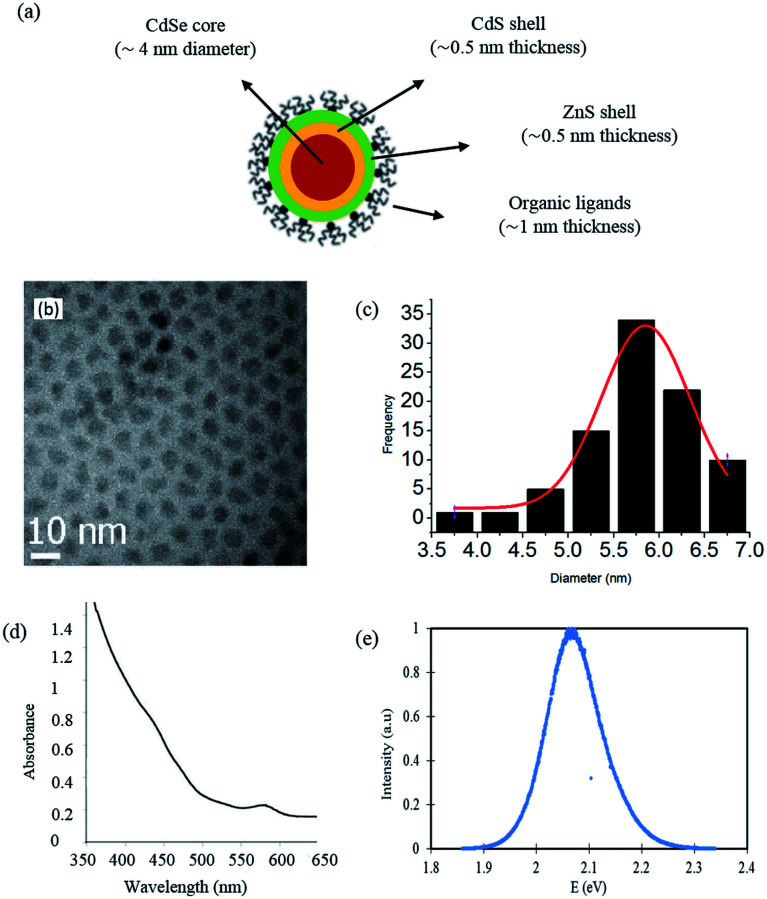
Selected CdSe/CdS/ZnS quantum dots: (a) schematic representation of the multi-shell structure; (b) TEM image (c) size distribution, (d) absorption spectrum and (e) photoluminescence spectrum.^29^

The strategy followed in the present work was to explore the capabilities of multi-shell structured CdSe/CdS/ZnS (see Fig. 1(a)) as pressure and temperature nanosensors in very thin films. The choice of these QDs was made based on the following considerations:

• The reported sensitivity of the CdSe-based QDs to temperature variations.^58^

• The important emission efficiency (or quantum yield) of CdSe/CdS/ZnS QDs (a value of 81% is reported in Protière *et al.*^29^). Such high efficiency is obtained thanks to the optimized core/shell structure with comparably low lattice mismatch between adjacent materials.

• The small size of these QDs compared to the range of film thicknesses found in EHD contacts (50–500 nm). Indeed, they have a mean diameter smaller than 10 nm, as it can be noticed on the histogram extracted from a Transmission Electron Microscopy (TEM) image and representing their size distribution (see Fig. 1(b) and (c)).

• The affinity of these QDs with apolar solvents which is ensured by the ligands. This property enables the formation of stable colloidal dispersions of the QDs within usual lubricants (*e.g.* hydrocarbons) which is a primary requirement to perform measurements within EHD contacts.

• The absorption and emission spectra of these QDs, found in the visible range (see Fig. 1(d) and (e)), allow them to be adapted to the instrumentation available with sensitive photodetector and to the systems that are used for studying EHD contacts (specimen transparent to visible radiation).

The choice of squalane was motivated by its rheological and optical properties (Table 1). Squalane is a model lubricant with well-known rheological properties,^39^ and it has a very weak absorption and no fluorescence response in the visible range. Thus, it will ensure no interference with the photoluminescence (PL) response of the QDs and, consequently, with the temperature and pressure measurements. A low concentration was chosen in order to prevent a change of the squalane rheological properties and also to minimize interactions between QDs that might modify the characteristics of the emission spectrum due to reabsorption processes.^62–65^

### Temperature and pressure calibration

**Fig. 2:**
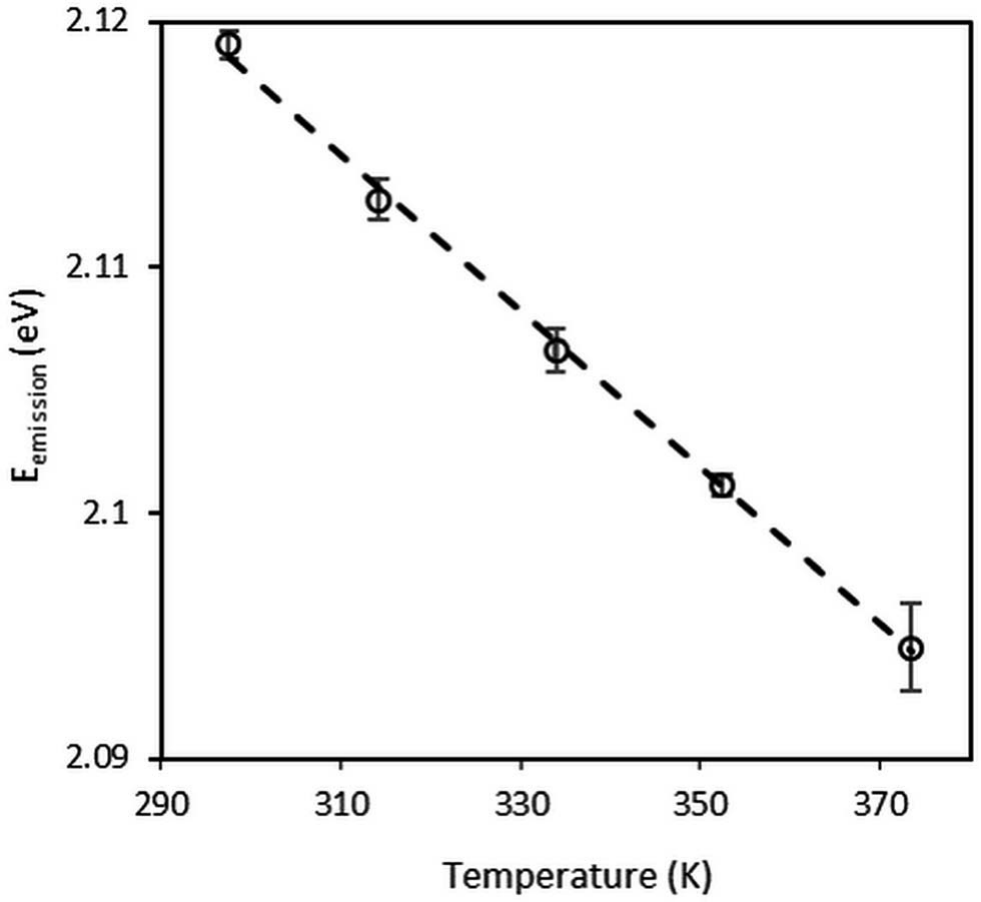
Temperature variation of the emission of CdSe/CdS/ZnS QDs dispersed in squalane at atmospheric pressure.

The temperature and pressure sensitivity to fluorescence energy of the QDs is needed to measure the local temperature and pressure in thin liquid films. The temperature dependence of the CdSe/CdS/ZnS QDs emission energy was measured in the temperature cell at ambient pressure. The temperature-induced emission energy shift of optical transition related to the direct energy gap is plotted in Fig. 2. At each temperature, four emission spectra were recorded at different positions at the mid-thickness of the sample. These measurements were used to estimate the mean emission energy and the standard deviation around the mean value, as displayed in Fig. 2. Symbols are the experimental points and the solid line is a linear fit.

It can be noticed that the emission energy decreases with increasing temperature. The experimental data have been fitted to the Varshni law (eqn (2)). The best-fit curve is obtained with *α* = 0.33 meV K^−1^ and *β* = 182 K. These values are consistent with: (i) the values of *α* = 0.28–0.41 meV K^−1^ and *β* = 181–315 K known in the literature for bulk semiconductor materials;^66^ (ii) the values *α* = 0.32 ± 0.02 meV K^−1^ and *β* = 220 ± 30 K reported by Valerini *et al.*^58^ for colloidal CdSe/ZnS QDs of 2.5 nm in radius embedded in an inert polystyrene matrix and for a temperature range between 45 and 295 K.

At high temperatures, in particular above the Debye temperature (*θ*_D_ ≈ *β*), a linear relation between band gap energy and temperature is expected (eqn (3)). Therefore, in Fig. 2, it can be considered that the emission energy decreases linearly with increasing temperature from ambient temperature to 373 K. The linear fit gives a coefficient of correlation of 0.99 and the temperature sensitivity *α*_1_ is calculated to be equal to −0.32 meV K^−1^. This result is fundamental for calibration purpose and nanothermometry application.

**Fig. 3:**
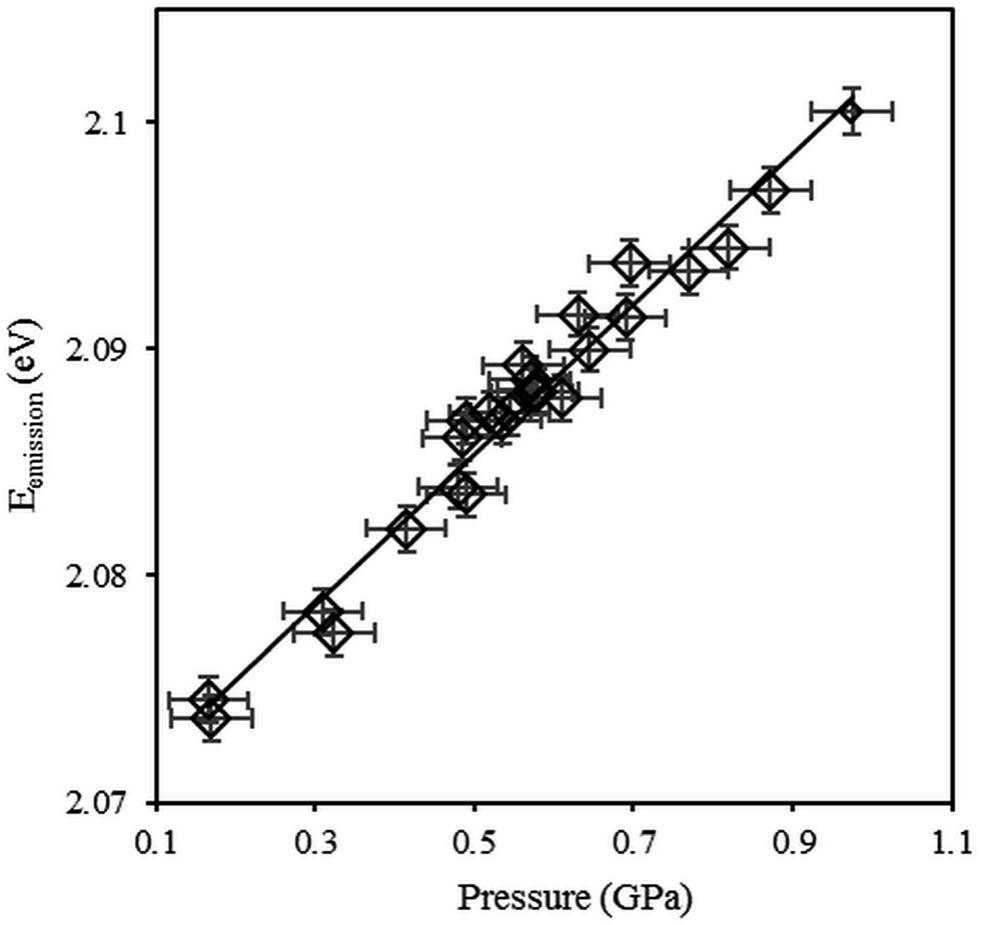
Pressure variation of the emission of CdSe/CdS/ZnS QDs dispersed in squalane at *T* = 296 K.

The pressure dependence of the CdSe/CdS/ZnS QDs emission energy at 296 K is shown in Fig. 3. At each pressure, four emission spectra were recorded at different positions in the diamond anvil cell, far from the gasket edge in order to avoid non-hydrostatic effects. Each spectrum was collected at a “z” position where the photoluminescence intensity reached a maximum. These measurements were used to estimate the mean emission energy and the standard deviation around the mean value as reported in Fig. 3. Symbols are the experimental points and the solid line is the linear fit. The pressure measurement uncertainty corresponds to the uncertainty of the ruby R1 luminescence line shift under pressure. In Fig. 3, spectra were recorded under increasing pressures. Other measurements were carried out with decreasing pressures (not shown here) with no evidence of hysteresis.

The emission energy is found to increase with increasing pressures. This behavior is in agreement with trends found in the literature for bulk materials^59,60^ and nanocrystals.^26,67,68^ For more quantitative analysis, emission energy variations with pressure were fitted using both linear and quadratic expressions (eqn (4) and (5)). The best quadratic fit yields the pressure coefficients *α*_2_ = 41.8 meV GPa^−1^ and *β*_2_ = −7.8 meV GPa^−2^. These values are consistent with the results reported in the literature.^59,60,68^ The best linear fit yields the pressure coefficient *α*_2_ = 33.2 meV GPa^−1^ with a correlation coefficient of 0.97. Thus, for pressure measurements the sensitivity of QDs to pressure will be considered as linear. Hereinafter, pressure sensitivity *α*_2_ will be used to refer to the linear pressure coefficient *α*_2_. For the sake of clarity, results at 323 K are not shown here. However, they do lead to the same conclusions: same pressure sensitivity with a shift to lower energies due to the temperature increase.

The calibrations have proven the potential of CdSe/CdS/ZnS QDs as nanosensors for both temperature and pressure measurements. Now that their sensitivity to fluorescence energy is well established, the linear relationships can be applied to measure temperature and pressure in thin liquid films.

### Shear-heating measurement under high shear rates

The first application deals with the measurement of shear heating (temperature rise due to viscous dissipation) inside a thin liquid film flowing in-between two parallel plates in a rheometer. Before carrying out the *in situ* measurement of temperature, it was important to ensure that the presence of the nanosensors in the fluid does not change its macroscopic behavior, in particular its rheological properties.

**Fig. 4:**
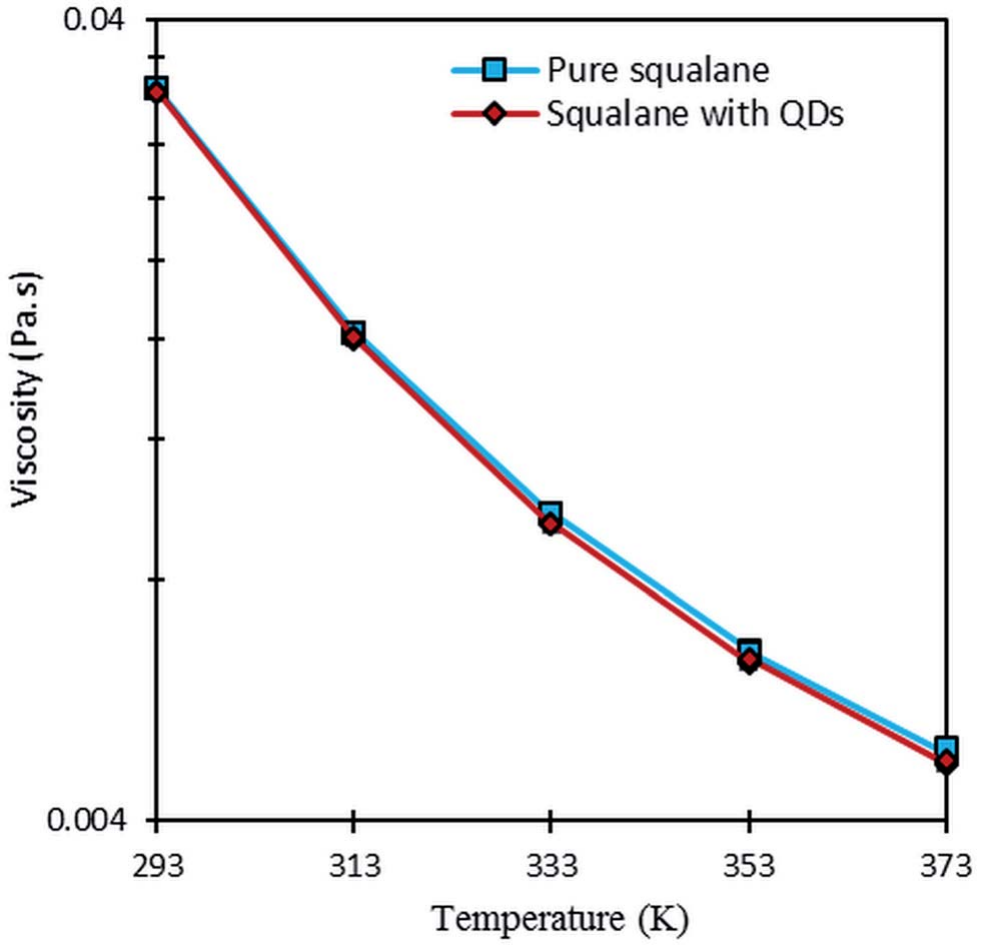
Comparison of the low shear viscosity of squalane with or without QDs at different temperatures.

Viscosity was measured at low shear rate and different temperatures for two fluids: (i) pure squalane; (ii) squalane with QDs. As expected from temperature–viscosity models in the literature (WLF model for instance),^39^ the low shear viscosity of both fluids varies nearly exponentially with temperature, as shown in Fig. 4. Furthermore, the influence of the QDs appears to be negligible given that the differences in viscosity are lower than 3%. This result opens the possibility of performing non-perturbing temperature measurements using the QDs, without introducing a noticeable change in the properties of the carrier fluid.

**Fig. 5:**
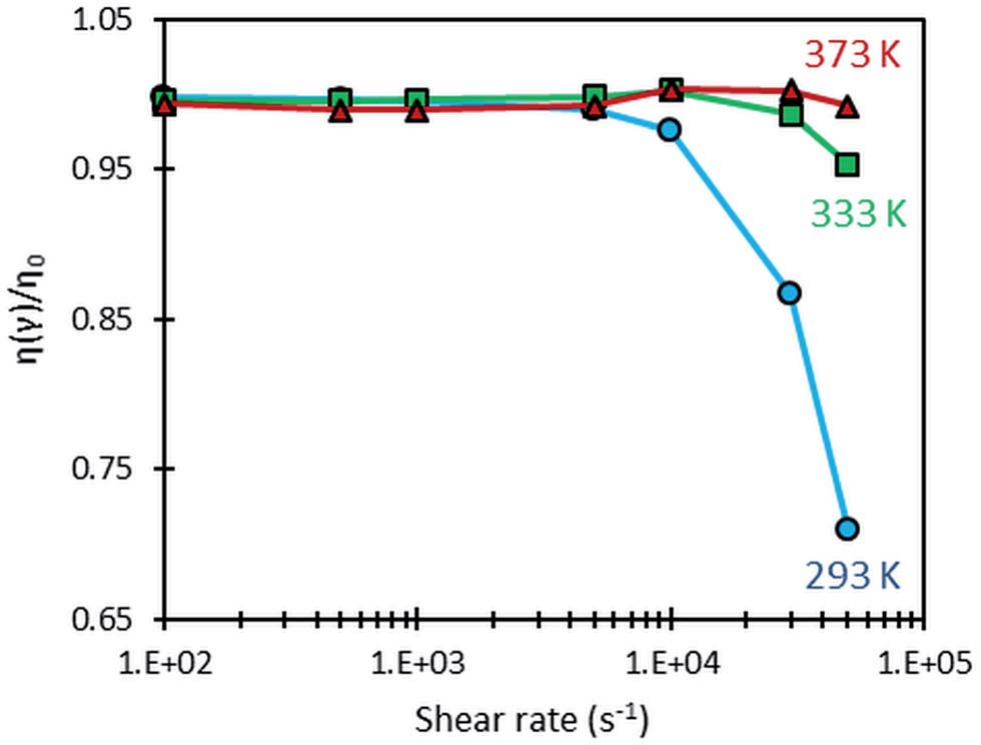
Variation of the relative viscosity of the squalane + QDs dispersion as a function of the shear rate at three temperatures.

A shear-heating effect was further induced in the optical rheometer by subjecting the sample to high values of shear rate. When this effect is significant, it is accompanied with an important drop in viscosity due to the temperature dependence of the fluid viscosity (as shown on Fig. 4). Fig. 5 shows the variation of the ratio *η/η*_0_, (where *η*_0_ is the low shear viscosity at *γ̇*= 10 s^−1^) measured with the optical rheometer as a function of shear rate at 293, 333 and 373 K. Shear heating, among other dependencies, is proportional to shear stress, which is viscosity time shear rate. When the latter is low enough, no shear heating occurs and the relative viscosity remains equal to one. This ratio is a convenient mean to quantify in a normalized and comparative way, the viscosity drop due to shear heating, whatever the considered temperatures. Clearly such self-heating occurs mainly at 293 K for shear rates higher than 10 000 s^−1^. Self-heating effect is actually more pronounced at low temperatures because of the larger temperature–viscosity dependence and the higher viscosity of the lubricant at these temperatures (as shown on Fig. 4), resulting in a more important viscous dissipation under shearing.

The occurrence of such heating can be further confirmed by using Nahme–Griffith number^40^ defined as:6
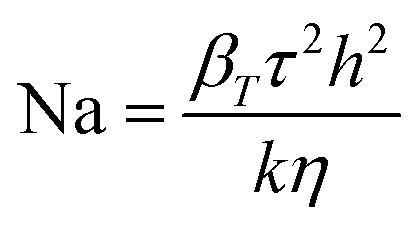


In this expression, *β*_*T*_ is the temperature–viscosity coefficient, *τ* the shear stress, *k* the liquid thermal conductivity and *h* the thickness of the flow. The values of *β*_*T*_ and *k* are given in Table 1. At 293 K, for a film thickness of 0.1 mm and for the shear rates of 30 000 and 50 000 s^−1^, Nahme–Griffith numbers of 0.13 and 0.36 are obtained, respectively. The latter value is larger than 0.3, which means that a significant thermal softening due to viscous heating occurs within the sample at these conditions.

By combining the viscosity drop observed in Fig. 5 and the viscosity–temperature dependence shown in Fig. 4, the temperature elevation at 293 K is estimated to be of about 3.4 and 8.9 K, for shear rates of 30 000 and 50 000 s^−1^, respectively.

**Fig. 6:**
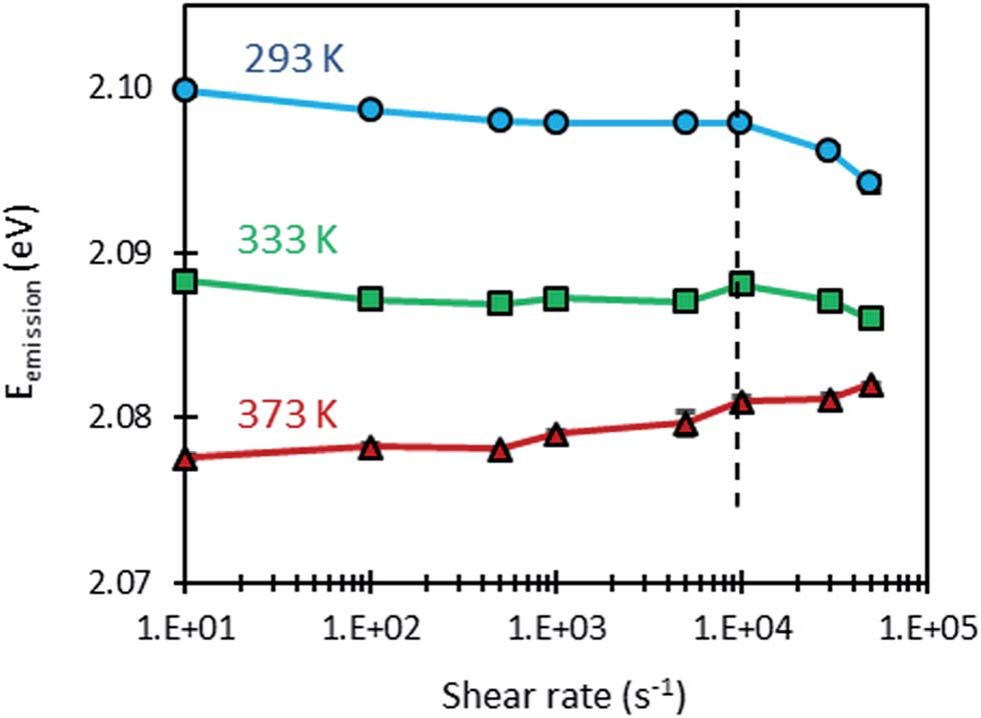
Variation of the emission energy of CdSe/CdS/ZnS QDs dispersed in squalane as a function of shear rate, at different temperatures.

In the same experiments as those that led to plot Fig. 5, the variation of the emission energy of the QDs with shear rate was measured at the same temperatures, as shown in Fig. 6. At each condition, measurements were repeated four times to estimate the mean emission energy and the standard deviation. It can be noticed that the error bars are remarkably small when compared with those obtained with the temperature cell. This can be explained by three factors: (i) the precise temperature regulation in the rheometer (Peltier-temperature-controlled system), (ii) the temperature homogenization due to the movement of the sample and (iii) the averaging of the emission spectrum over a large sample volume during the rotation of the latter.

Furthermore, Fig. 6 shows that the response of the QDs is almost unaffected by the dynamic conditions induced by shear rate for values below 10 000 s^−1^. Consequently, the values of emission energy obtained at different temperatures and shear rates smaller than 10 000 s^−1^ were used to compute the temperature sensitivity of the QDs under dynamic conditions: *α*^Dynamic^_1_. As shown in Table 4, dynamic temperature sensitivities are close to those obtained under static conditions. These different findings demonstrate that the temperature calibrations made under static conditions can be used for measurements under dynamic conditions.

**Table 4:** Temperature and pressure sensitivities of CdSe/CdS/ZnS QDs dispersed in squalane, under static and dynamic (temperature only) conditions

*α*_1_ (meV K^−1^)	*α* ^Dynamic^_1_ (meV K^−1^)	*α*_2_ (meV GPa^−1^)
−0.32 ± 0.01	−0.28 ± 0.02	33.2 ± 3

**Fig. 7:**
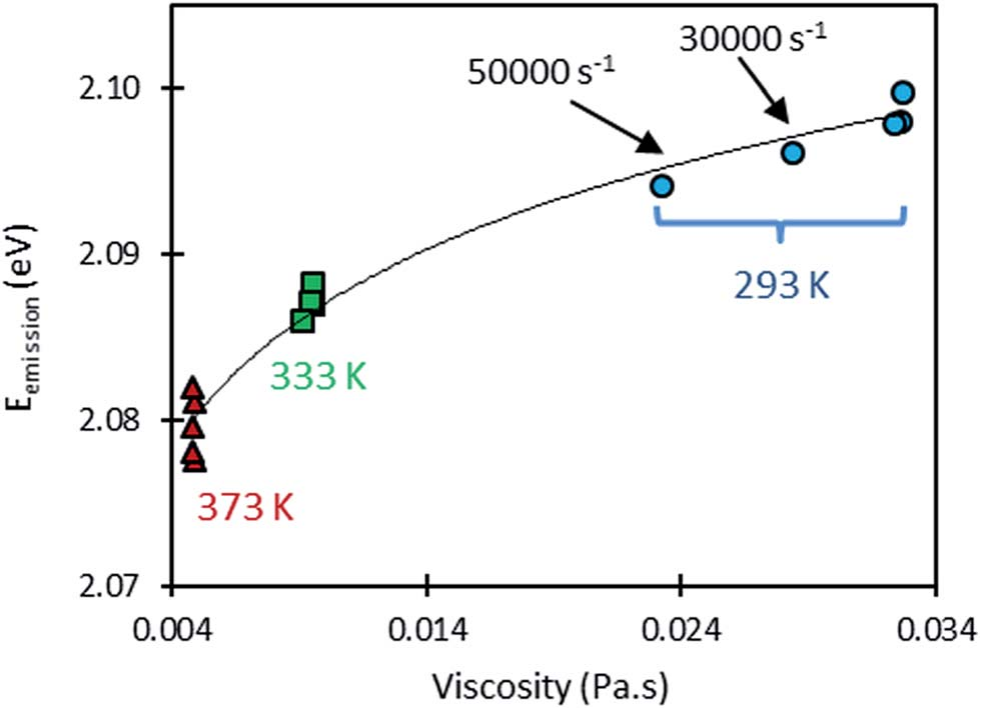
Variation of the emission energy (dots) of CdSe/CdS/ZnS QDs dispersed in squalane as a function of the measured viscosity for five imposed shear rates (from 10 s^−1^ to 50,000 s^−1^) at three different temperatures (solid line is the logarithm fit to the experimental results).

When considering shear rates larger than 10 000 s^−1^, however, some variations in emission energy can be observed. High shear rates can induce a viscosity drop as a result of self-heating as shown in Fig. 5, but in the same time, this heating decreases the emission energy as a result of the sensitivity of the QDs to temperature. Thus, it is informative to examine the variation of the emission energy with viscosity (see Fig. 7). A logarithmic law was used to fit this variation. This choice is justified by the linear variation of emission energy and the nearly exponential variation of viscosity with temperature. At the temperature of 293 K however, the points (*E*_g_, *η*) measured at high shear rates (30 000 and 50 000 s^−1^) are spaced from the others points obtained at smaller shear rates. Remarkably, these points follow well the fitting curve linking the measurements made at 293 K, 333 K and 373 K. This observation confirms that the shift in the emission energy (to lower values) is correlated to the drop in viscosity as a result of the shear-induced heating at high shear rates.

Based on the above-mentioned findings, using QDs fluorescence seems to be adequate for the estimation of the temperature rise in the sample subjected to high shear rate at 293 K. By using the sensitivity of the QDs obtained under static conditions (*i.e. α*_1_ = −0.32 meV K^−1^), a temperature rise of 2.8 and 8.9 K can be deduced for the shear rates 30 000 and 50 000 s^−1^, respectively. Table 5 compares the temperature rises as obtained from the viscosity drop on one hand, and those obtained from the variation of emission energy on the other hand. The fair agreement between the two approaches for temperature rise estimation confirms the potential of these QDs for temperature probing under dynamic conditions and for further applications like EHD lubrication.

**Table 5:** Comparison of the temperature rises calculated from the energy and viscosity decreases at 293 K

Shear rate (s^−1^)	Temperature rise (K)	
	From energy decrease^a^	From viscosity decrease
30 000	2.8	3.4
50 000	8.9	8.9

a *α*_1_ = −0.32 meV K^−1^ (measured in the calibration study).

### Pressure measurement in EHD contacts

The second application of QDs as nanoprobes deals with the measurement of pressure at the center of an EHD contact. As shown before, the presence of nanosensors in squalane does not change its rheological properties at ambient pressure. It is then expected that the film thickness generated by the suspension of QDs in squalane to be the same as the one obtained with pure squalane.

For each of the operating conditions detailed in Table 3, various spectra were recorded along the rolling direction (*x* direction) from the inlet to the center of the contact. At the contact inlet, five spectra were recorded at the following dimensionless coordinates *x/a* from the contact center: −4, −3, −2, −1.5, and −1.25. Three spectra were also recorded at the center of the contact and in its immediate vicinity (±10 μm) to measure the pressure at the contact center area.

**Fig. 8:**
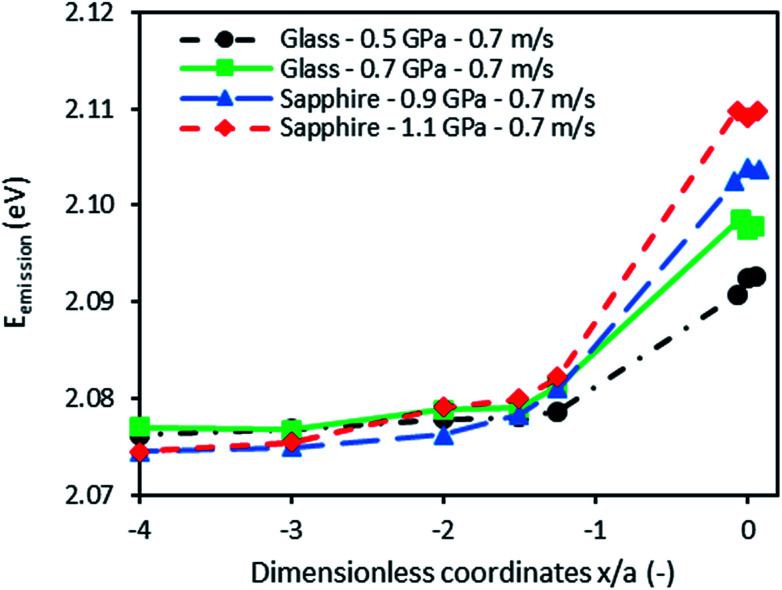
Emission energy variation from the inlet to the center of the contacts at 293 K.

Fig. 8 shows the emission energy recorded along the rolling direction at different Hertzian pressures (from 0.5 to 1.1 GPa) in pure rolling and at an entrainment speed of 0.7 m s^−1^. The fluorescence energy increases at the inlet of the contact (for *x*/*a* ≥ −2). This observation is consistent with the well-known pressure rise predicted by the EHL theory. An increase of emission energy is equivalent to an increase of pressure according to the calibration results. Thus two distinct zones may be distinguished:

• The inlet of the contact where the pressure is built up (at a coordinate above *x/a* ≥ −2) due to the converging geometry.

• The high pressure zone at the contact center where the energy and thus the pressure are found at their maximum values.

In order to quantify the pressure at the contact center, the emission energy shift between the contact center and the inlet zone far from the former was divided by the linear pressure sensitivity, *α*_2_, determined from the static pressure calibration:7
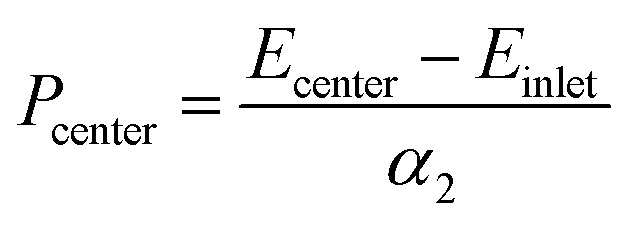
where *E*_inlet_ is the mean energy measured at *x*/*a* = −4 and *x*/*a* = −3. At these coordinates, the fluid is considered to be at atmospheric pressure, which was confirmed by the numerical simulations.

**Fig. 9:**
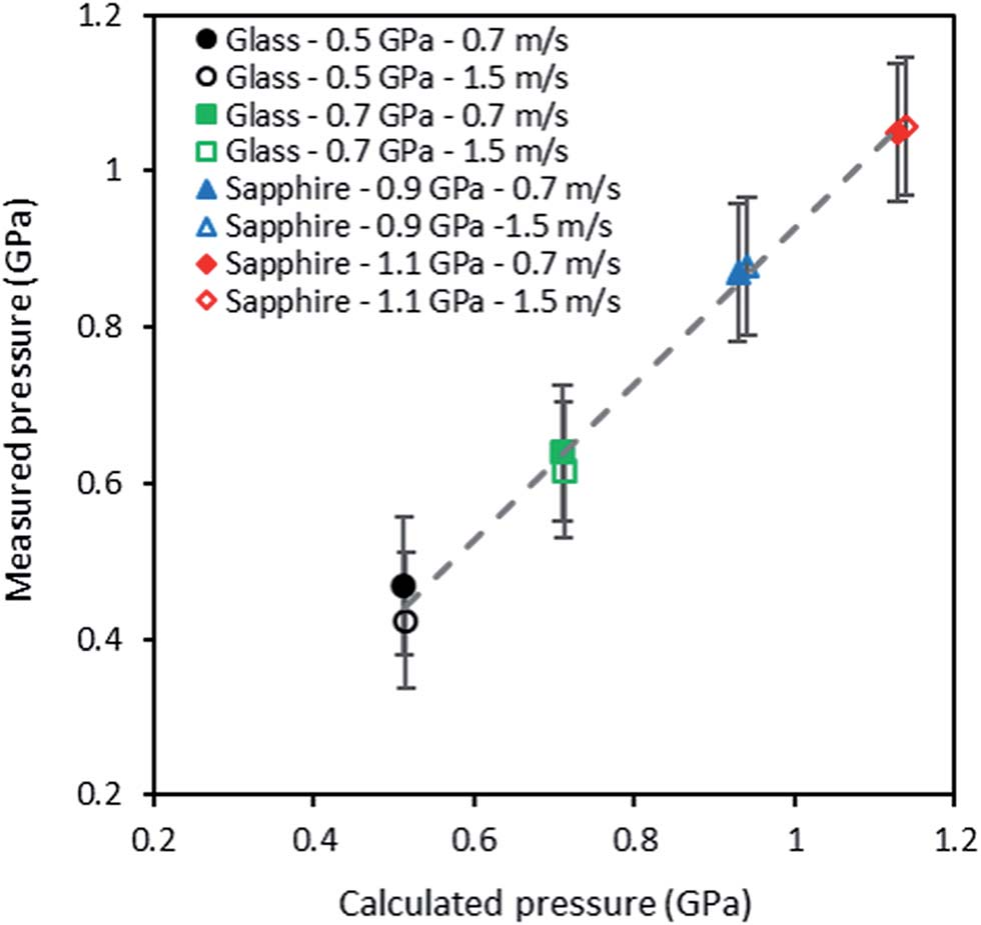
Calculated pressures *versus* measured pressures obtained *in situ* through the photoluminescence response of QDs dispersed in squalane at 293 K, for two entrainment speeds and four contact pressures.

The experimental and numerical values of pressure are reported in Fig. 9, for two rolling speeds (0.7 and 1.5 m s^−1^) and four Hertzian pressures (0.5, 0.7, 0.9 and 1.1 GPa). A linear regression was used to fit experimental values with numerical ones: a slope of 0.99 was found with a coefficient of correlation of 0.99. In spite of the rather large uncertainty on pressure measurements inherent to the ruby R1 fluorescence line (*i.e.* not to the *in situ* technique), these results show a very good agreement between experimental values and numerical predictions.

## Conclusion

This work has demonstrated the feasibility of measuring temperature and pressure in very thin liquid films, down to a thickness of about 200 nm for the later. The measurement technique is based on the dependence of photoluminescence energy of quantum dot nanoparticles with temperature and pressure. The QDs sensitivities have been determined under static conditions. Linear relationships have been found between fluorescence energy temperature and pressure in the domains of 293–373 K and 0–1.1 GPa, respectively.

Temperature has been measured in a thin liquid film subjected to shear heating between two parallel plates in an optical rheometer. Pressure has been measured within an EHD point contact, under pure rolling and isothermal conditions. In both cases, the experimental values have been compared with theoretical or numerical predictions. The very good agreements that resulted from these comparisons allowed a validation of the experimental methodology and proved its high potential to probe with confidence highly confined liquid films, as those occurring in EHD contacts. Even if the authors had a strong motivation for the application of this technique to the study of lubricant thin films, it clearly appears that other fields could benefit from it, as for example (but not limited to) micro or nanofluidics, nanoelectronics, photonics, biophysics, biomedicine *etc.*

However, for simultaneously mapping temperature and pressure in a highly confined liquid, some challenges still have to be overcome. In particular, fluorescence energy has been shown to be dependent on both temperature and pressure. Therefore, to measure simultaneously these two quantities, it will be necessary to uncouple the temperature and pressure dependencies of the QDs, which represents the next challenge to achieve.

## Conflicts of interest

There are no conflicts to declare.

## List of symbols

*a*
: Contact radius (m)
*C*_p_
: Heat capacity (J kg^−1^ K^−1^)
*E*
: Young modulus (GPa)
*E*_center_
: Emission at the contact center (eV)
*E*_emission_
: Emission energy (eV)
*E*_inlet_
: Emission at the contact inlet (eV)
*E*_g_
: Bandgap energy (eV)
*E* ^B^_g_
: Bulk contribution to the bandgap energy (eV)
*E* ^Q^_g_
: Quantum contribution to the bandgap energy (eV)
*h*
: Film thickness (m)
*h*_c_
: Central film thickness (m)
*k*
: Thermal conductivity (W m^−1^ K^−1^)
Na: Nahme–Griffith number
*P*
: Pressure (GPa)
*P*_center_
: Pressure at the contact center (GPa)
*R*
: Radius of the QD (nm)
*R*
: Radius of the rheometer rotating disc (mm)
*T*
: Temperature (K)
*u*_e_
: Entrainment velocity (m.s^−1^)
*w*
: Load (N)
*x*
: Coordinates (—)
α*: Reciprocal asymptotic isoviscous pressure (GPa^−1^)
*α*
: Constant in Varshni law (meV K^−1^)
*α*_1_
: Constant in the linear approximation of Varshni law (meV K^−1^)
*α* ^Dynamic^_1_
: Dynamic temperature sensitivity (meV K^−1^)
*α*_2_
: Constant in the linear and quadratic approximation of *E*_g_ variation with pressure (meV GPa^−1^)
*β*
: Constant in Varshni law (K)
*β*_2_
: Constant in the quadratic approximation of *E*_g_ variation with pressure (meV GPa^−2^)
*β*_T_
: Temperature–viscosity coefficient (K^−1^)
*β*_1/*T*_
: Temperature–viscosity coefficient (K)
*η*
: Measured viscosity (mPa s)
*η*_0_
: Low shear viscosity (mPa s)
*θ*_D_
: Debye temperature (K)
*ν*
: Poisson ratio (—)
*ρ*
: Density (kg m^−3^)
*τ*
: Shear stress (MPa)
*ϕ*_*T*_
: Film thickness thermal reduction coefficient according to Cheng (—)

## Supplementary Material
